# Perspectives on magnetic reconnection

**DOI:** 10.1098/rspa.2016.0479

**Published:** 2016-12

**Authors:** Ellen G. Zweibel, Masaaki Yamada

**Affiliations:** 1 Departments of Astronomy and Physics, University of Wisconsin-Madison, Madison, WI, USA; 2 Princeton Plasma Physics Laboratory, Princeton University, Princeton, NJ, USA

**Keywords:** magnetic fields, magnetic reconnection, particle heating, particle acceleration, plasma physics

## Abstract

Magnetic reconnection is a topological rearrangement of magnetic field that occurs on time scales much faster than the global magnetic diffusion time. Since the field lines break on microscopic scales but energy is stored and the field is driven on macroscopic scales, reconnection is an inherently multi-scale process that often involves both magnetohydrodynamic (MHD) and kinetic phenomena. In this article, we begin with the MHD point of view and then describe the dynamics and energetics of reconnection using a two-fluid formulation. We also focus on the respective roles of global and local processes and how they are coupled. We conclude that the triggers for reconnection are mostly global, that the key energy conversion and dissipation processes are either local or global, and that the presence of a continuum of scales coupled from microscopic to macroscopic may be the most likely path to fast reconnection.

## 1. Introduction

Magnetic fields are ubiquitous. Planets, stars, the tenuous interstellar gas in galaxies and the even more tenuous plasma in galaxy clusters are magnetized: magnetic fields may well pervade the entire cosmos. Magnetic fields in space plasmas directly affect terrestrial life. Magnetic confinement of hot plasma for controlled nuclear fusion is one of the most ambitious and promising goals of energy science and technology. The properties of these magnetized plasmas span a vast range, but there are a few basic processes that occur in almost all of them. One of the most important is magnetic reconnection.

Magnetic reconnection is the topological rearrangement of the magnetic field in a plasma on a time scale faster than allowed by microscopic forms of dissipation. Although this definition makes no reference to magnetic energy release, reconnection was originally conceived as a way to power storms and flares in space plasmas and on the Sun. The relative importance of energy conversion and topological change as defining properties of reconnection, and the extent to which they are linked, are key issues in reconnection research to which we will return in this article.

Evidence that reconnection releases energy comes in the form of fast particles and fast outflows associated with solar/stellar flares, magnetospheric substorms and laboratory sawtooth crashes. Evidence for topological rearrangement in natural plasmas is largely morphological, while in laboratory and space plasmas it can come directly through *in situ* measurements of the magnetic configuration and through measurements of plasma and energy confinement. Reconnection affects plasma dynamics, energetics and transport. It couples global and local scales, is deeply implicated in plasma self-organization, and is necessary for magnetic field amplification by a dynamo.

In the recent two decades, collaborative reconnection research between the laboratory and space plasma physics communities has increased significantly, and it is now clear that multi-disciplinary approaches to studying these plasmas are fruitful beyond direct intercomparison of their properties. While the dimensionless parameters of natural plasmas are often beyond the reach of numerical simulations or laboratory experiments, the experiments themselves can often be simulated, resulting in validated computational and theoretical tools which can be applied to natural plasmas. This often yields new insights into both the laboratory and natural systems.

Much reconnection research is organized around four key problems:

*The rate problem*. Reconnection rates inferred from observations, such as the morphological changes and onset of hard X-ray emission associated with solar flares, are often much faster than predicted by theory. Identifying mechanisms for fast reconnection, particularly under astrophysical conditions, has been a major theme in reconnection research.
*The trigger problem*. Understanding what determines the amount of energy that can be stored in a magnetic field and what triggers its release is necessary for controlling laboratory plasmas and predicting reconnection in natural plasmas.
*The energetics problem*. A theory of how energy is apportioned between bulk outflows, ion heating, electron heating and non-thermal energetic particles is lacking, but necessary for interpreting diagnostics of reconnection.
*The interplay of scales problem*. This problem is particularly acute in astrophysics, where microscales such as the particle collisional mean free paths and gyroradii are often many orders of magnitude less than the global scales. While magnetic field lines break on microscopic scales, energy is stored and the plasma responds on global scales. Understanding how the large and small scales are coupled is required for an integrated view of reconnection.

Although we discuss recent progress on all of these problems, we emphasize the latter two, especially as we contemplate future prospects. There has been notable progress on the rate problem, while the trigger problem is to some degree addressed by the scale problem. On the other hand, the availability of new laboratory, spacecraft and simulation tools has created a frontier in reconnection energetics. Meanwhile, the increasing need for accurate, predictive theories of reconnection for both interpretation of astrophysical data and simulation of astrophysical systems demands understanding how local and global scales are coupled.

In order to convey the issues in play in the scale problem, we have taken a single-fluid magnetohydrodynamic (MHD) approach versus a kinetic or two-fluid approach. In §2, we set up the problem as an MHD problem and show how the rate, trigger and scale issues arise. In §3, we address global energy conversion and dissipation arising from reconnection. In §4, we discuss mechanisms for fast reconnection in MHD. In §5, we undertake a detailed examination of the reconnection layer in the context of two-fluid analysis, and in §6 we describe a quantitative experimental study of reconnection energetics. We conclude in §7 that the triggers for reconnection are mostly global, that the key energy conversion and dissipation processes are either local or global, and that the presence of a continuum of scales coupled from microscopic to macroscopic may be the most plausible path to fast reconnection.

Reconnection research covers plasmas of many types, including weakly ionized, radiation dominated, electron–positron pair and relativistic. Many interesting features emerge in these regimes, but we will mention them only insofar as they bring out basic physics points; we also restrict ourselves by and large to the simpler quasi-2D geometries. This is not a comprehensive review of reconnection, nor is the bibliography comprehensive. The reader seeking such material should consult [1–3], or [4] for reconnection in general, [5] for reconnection in extreme environments, [6,7] for reviews of reconnection in astrophysics that emphasize collisionless effects and turbulence, respectively, [8] for a review emphasizing the role and formation of plasmoids and [9] for a review of recent laboratory results. Likewise, although reconnection is studied in a number of dedicated laboratory experiments and observed in many plasma confinement experiments, the experimental results we discuss here will primarily be those obtained with the magnetic reconnection experiment (MRX) at the Princeton Plasma Physics Laboratory [4,9,10] because we believe that detailed description of specific results will explain the key physical process more clearly. In appendix A, we present table 1, which summarizes the major dedicated laboratory devices which were built since 1970 and are dedicated to the study of magnetic reconnection, including the recent four major experiments [4], TS-3/4 in UTokyo [11], MRX [9], SSX at Swarthmore [12] and VTF at the Massachusetts Institute of Technology [13].

## 2. Theoretical formulation of magnetic reconnection based on magnetohydrodynamics

The evolution of a magnetic field **B** is determined by Faraday’s law

2.1 $$\frac{\partial \text{B}}{\partial t}=-c\nabla \times \text{E},$$

where **E** is the electric field (here as elsewhere we use Gaussian cgs units). Formally, if **E** can be written as

2.2 $$\text{E}=-\frac{{\text{u}}_{B}\times \text{B}}{c}+\nabla \Phi ,$$

then **u**_*B*_=*c*(**E**−**∇***Φ*)×**B**/*B*^2^ can be said to be the velocity of the field lines and to describe magnetic evolution with fixed topology [14,15]. Equation (2.2) breaks down at magnetic nulls (*B*≡0) and on closed field lines, where *Φ* is undefined. Thus, reconnection defined from a topological perspective as a situation in which it is impossible to globally identify and follow field lines can only occur where **E** cannot be written in the form of equation (2.2). We will see that departures from equation (2.2) sometimes but not always imply significant transfer of magnetic energy to other forms.

In plasma physics, **E** is found from Ohm’s law.^1^ If the plasma is collisional, it can be treated in the MHD approximation, i.e. as a conducting fluid with a well-defined velocity **u**. In this case, Ohm’s law is

2.3 $$\text{E}=-\frac{\text{u}}{c}\times \text{B}+\frac{\text{J}}{\sigma },$$

where *σ* is the electrical conductivity and **J** is the current density.

Equations (2.1) and (2.3) together with Ampere’s law (neglecting the displacement current) lead to the magnetic induction equation

2.4 $$\frac{\partial \text{B}}{\partial t}=\nabla \times (\text{u}\times \text{B})+\eta \nabla^{2}\text{B},$$

where *η*≡*c*^2^/4*πσ* is the magnetic diffusivity, which we have assumed constant for simplicity.

The relative importance of diffusion and advection on *global* scales *L*∼|∇|^−1^ in determining the evolution of **B** is measured by the magnetic Reynolds number, Rm,

2.5 $$\text{Rm}\equiv \frac{Lu}{\eta }.$$

In moderately large laboratory plasmas Rm is typically of order 10^4^–10^8^, in the Sun Rm∼10^8^–10^14^, while in the interstellar medium of galaxies Rm∼10^15^–10^21^.

If Rm is large, it is tempting to drop the resistive term in equation (2.4). This is the ideal MHD approximation and corresponds to equation (2.2) with **u**_*B*_=**u**. Equation (2.4) can then be integrated to give the magnetic field **B**(**x**,*t*) in terms of its initial value and the fluid displacement

2.6 $$\text{B}(\text{x}({\text{x}}_{0},t),t)=\text{D}\cdot {\text{B}}_{0}({\text{x}}_{0})|D{|}^{-1},$$

a result known as the Cauchy formula. In equation (2.6), **x**(**x**_0_,*t*) is the position at time *t* of a fluid element that was at **x**_0_ at *t*=0, **B**_0_ is the magnetic field at *t*=0, **D** is the deformation matrix

2.7 $$\text{D}\equiv \frac{\partial \text{x}}{\partial {\text{x}}_{0}}$$

and |*D*| is its determinant. Equation (2.6) implies that fluid elements that are magnetically connected at one time are connected for all times. The magnetic field is thus said to be frozen to the plasma. This result is equivalent to Alfvén’s theorem.

Despite the large values of Rm in natural plasmas, flux freezing must break down; magnetic connectivity cannot be preserved over cosmic time. Three interrelated concepts have been introduced to explain how the frozen flux constraint can be broken:

(i) Boundary layers form in which **J** is very large, leading to locally small Rm and local resistive diffusion with the field frozen in almost everywhere else. This scenario predicts the location and onset conditions for reconnection, which are well confirmed by laboratory experiments.
(ii) The MHD model breaks down at small scales and/or low collisionality, introducing non-resistive effects which break the field lines. Plasma effects have been measured in laboratory experiments and in space plasmas.
(iii) The elements of **D** can grow without bound if neighbouring fluid elements rapidly separate, which is the case if the fluid motion is turbulent. If the motion of fluid elements is not well defined, the magnetic field cannot be said to be frozen to the fluid. There is currently no laboratory evidence for this process.

**Figure 1.**
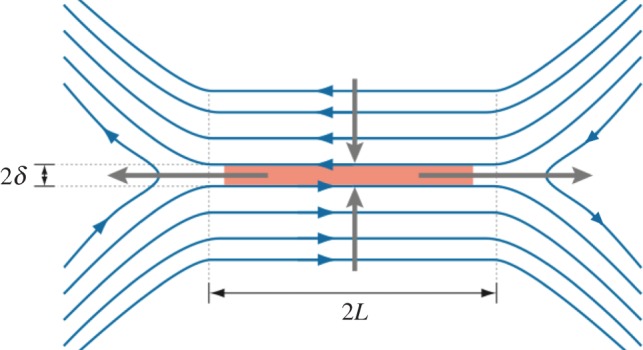
Magnetic field geometry for the Sweet–Parker model. Oppositely directed field lines are brought together and reconnect in a diffusion layer (orange). The plasma is heated by Ohmic dissipation at the diffusion region and accelerated by the pressure gradient and the tension force. The field line diffuses over the half-width of the diffusion layer, *δ*, which is much smaller than the system size, 2*L*.

One of the first models of reconnection was the Sweet–Parker model [16,17]. This model, which is depicted in figure 1, is of the boundary layer type. It is steady state and two-dimensional (2D). The length and width of the boundary layer are 2*L* and 2*δ*, respectively. Plasma, to which the magnetic field is frozen and which is assumed to be incompressible, flows into the boundary layer with speed *v*_in_. Flux freezing breaks down at the magnetic X-point, where the field line velocity **u**_*B*_ introduced in equation (2.2) deviates from the plasma velocity **u** and becomes undefined. The tension force due to the sharp bend in the reconnected field lines drives a plasma flow at the Alfvén speed $v_{A}\equiv B/\sqrt{4\pi \rho }$ away from the X-point. From conservation of mass, *ρv*_in_*L*=*ρv*_*A*_*δ*, and uniformity of the electric field, *v*_in_*B*/*c*=*J*/*σ*=*ηB*/*δc*, it follows that

2.8 $$v_{\mathrm{in}}=v_{A}S^{-1/2}$$

and

2.9 $$\delta =LS^{-1/2},$$

where *S* is the Lundquist number

2.10 $$S\equiv \frac{Lv_{A}}{\eta }=\text{Rm}\frac{v_{A}}{u}.$$

The relationship

2.11 $$\frac{v_{\mathrm{in}}}{v_{\mathrm{out}}}=\frac{\delta }{L}$$

implied by equations (2.8) and (2.9) is a consequence of the equation of continuity and is fundamental to reconnection theory.^2^ It can be shown from equations (2.8) and (2.9) that the kinetic energy flux in the outgoing Alfvénic jets and the energy flux in Ohmic heating are approximately the same. In §6, we will describe the experimentally measured energy fluxes in a laboratory reconnection layer.

The Lundquist number *S*, like Rm, is typically large in laboratory, space and astrophysical plasmas. This makes the inflow velocity *v*_in_ very small and the reconnection rate very low. For typical solar coronal loop parameters, for example, the reconnection time is of the order of 1 year instead of the observed rise time of solar flare emission, which is less than a few minutes. It was, therefore, soon recognized that the Sweet–Parker reconnection could not completely describe the energy release in solar flares.

The Sweet–Parker model, being steady state, does not address reconnection onset or its cause. Furth *et al.* [19] identified a class of resistive instabilities known as tearing modes, which allow the magnetic energy of a system to be reduced by motions that violate the frozen flux constraint. Linear tearing mode eigenfunctions resemble the Sweet–Parker reconnection in that there is an outer region where **E** is inductive and an inner region where it is resistive. In the resistive region, MHD restoring forces must be weak. This requires that the inductive electric field term (**k**⋅**B**_0_)**u**_1_≡0, where **k**, **B**_0_ and **u**_1_ are the perturbation wavenumber, equilibrium magnetic field and velocity perturbation, respectively. In doubly periodic systems such as axisymmetric tori where **k** is quantized, this condition can generally only be satisfied on particular surfaces known as rational surfaces (in cylindrical systems, the rational surfaces occur where the *q* parameter defined in footnote 3 is rational). The resistive inner layer thickness *δ*_T_ and growth rate *γ*_T_ of the tearing mode both scale as fractional powers of *S* (*δ*_T_∝*S*^−2/5^, *γ*_T_∝*S*^−3/5^ for a slab), and thus are slow in the same sense as the Sweet–Parker reconnection. We will refer to reconnection as **fast** if the rate is independent of *S* in the MHD regime or independent of a microscale in the two-fluid or kinetic regime. However, the mechanisms responsible for fast reconnection vary with the plasma model, and in cases where the reconnection rate can be measured reliably, fast MHD reconnection is generally slower than fast two-fluid or kinetic reconnection by about an order of magnitude.

The Sweet–Parker reconnection and resistive tearing are multi-scale processes. While the conditions leading to reconnection and its global impact are macroscopic, the field lines break on scales that are smaller than the global scale by a fractional power of *S*. In §5, we discuss the conditions under which the field line breaking scale is so small that resistive MHD does not apply.

Non-thermal particle acceleration in reconnection is further evidence for kinetic processes. The *γ*-ray flares observed from the Crab nebula (recently reviewed in [20]) could be a particularly intriguing case. The arguments for acceleration by reconnection are strong but indirect. Electrons accelerated by an MHD flow with [*E*]/[*B*]∼*v*/*c*<1 cannot radiate synchrotron photons more energetic than about 160 MeV [21], a value derived by balancing the radiative loss rate against the maximum possible acceleration rate. Yet, the Crab flares extend to the GeV range, suggesting [*E*]/[*B*]>1. The rise times of the flares are hours, suggesting a small source and a fast energization mechanism. These points, taken together, suggest fast magnetic reconnection [22–24].

## 3. Global effects

It appears that, if the magnetic energy of a low *β*≡8*πP*/*B*^2^ global MHD equilibrium state would be lowered by a reorganization of plasma topology, reconnection will take place. Likewise, reconnection will stop if it no longer lowers the total magnetic energy. It is recognized that global reconnection or magnetic self-organization phenomena almost always occur unsteadily or impulsively at a fast rate following a long energy build-up phase. For example, the high-energy electrons generated during the global reconnection phenomena observed in solar flares require up to 50% of the magnetic energy accumulated before the reconnection events.

Important progress has been made on global reconnection physics by studying relaxation phenomena in laboratory fusion plasmas. A large-scale MHD instability driven by global boundary conditions often produces a current layer on the mode rational surface, inducing magnetic reconnection. Magnetic energy stored over a long period of time in a plasma system is then released, globally driving the plasma to a relaxed state. Strong acceleration of ions and electrons occurs during this relaxation process. In tokamak discharges, reconnection often occurs quite suddenly after a slow evolution of plasma equilibrium and magnetic flux build-up. Generally, the flux build-up phase is significantly longer than the reconnection time. This creates a sawtooth-shaped evolution of the central electron temperature. Whereas prior to reconnection electrons are well confined by coaxial magnetic flux surfaces, after reconnection the field develops regions of stochasticity that result in loss of plasma confinement [4]. This ‘sawtooth crash’ is a good example of how evolution of the global plasma configuration forces local reconnection.

Tokamaks are so-called high *q* plasmas because of their low field line pitch.^3^ Sawtooth events are also observed in low-*q* pinch discharges such as the spheromak and the RFP (reversed-field pinch) [3]. As in tokamaks we observe a slow flux build-up phase through slow reconnection and a fast reconnection/relaxation phase. In the former phase the current density in the centre core gradually increases, while in the latter an impulsive current profile flattening occurs with reconnection.

Reconnection does not always disrupt confinement. The toroidal field reversal in the RFP is due to reconnection and can be understood as relaxation to the minimum energy state with fixed global magnetic helicity $H\equiv \int_{V}(\text{A}\cdot \text{B})\;dV$ in accordance with Taylor’s conjecture [25,26]. Generally, reconnection occurs in the resonant flux surfaces in the plasma core and, under some conditions, at the edge. In some cases, two unstable tearing modes in the core region are observed to couple each other to nonlinearly drive reconnection at the resonant surface of a linearly *stable* third mode in the outer plasma edge region [27]. This is an important example of driven reconnection.

All three global aspects of reconnection—as a disruptor, as an agent of relaxation and as a driver of further reconnection—are conjectured to occur on the Sun; the first and third in flares and mass ejections, the second as a more quiescent coronal heating mechanism [28,29]. Thus, it appears that global magnetic self-organization phenomena in laboratory sawtooth crashes, plasma relaxation, solar flares and coronal heating are mediated at least in part by reconnection.

The MRX experiment provides examples of how global conditions affect the details of the reconnection process itself. In MRX, a large downstream pressure was found to slow both the outflow in the reconnection layer and the reconnection rate, demonstrating the importance of boundary conditions [30]. It was also found that, with the same plasma parameters, the reconnection rate decreases with increasing distance between flux cores or equivalently with system size [31]. The reduced reconnection rates in larger systems were attributed to longer current sheets. In addition to this dependence on the system size, the current sheet length *L* was found to depend on the effective resistivity, *η*_eff_≡|*E*/*J*|. The exact cause of the enhanced resistivity above Coulomb was not determined at the time. For a given system size, the current sheet length anti-correlates with the effective resistivity, i.e. the current sheet length varies inversely with resistivity.

Recently, the effects of external forcing on driven reconnection have been studied in MRX. A simple model based on a feedback loop has been developed to explain the details of the linear and overdriven scaling regimes. It is found that driven magnetic reconnection may be modelled as an interplay between the external forcing and the dynamics of the current sheet region. By investigating the effects of external forcing in MRX, linear and overdriven regimes are identified. When the external forcing is applied at a slower rate than the flux penetration time scale of the current layer, the reconnection speed is proportional to the external driving rate; thus, the linear response regime. By contrast, in the overdriven regime, the incoming magnetic flux cannot penetrate into the reconnection layer at the rate prescribed by the coils. As a result, the reconnection rate saturates at a value determined by the penetration time scale, namely the intrinsic reconnection speed determined by the local dynamics [32].

## 4. Description of fast reconnection in magnetohydrodynamics

The slow reconnection predicted by Sweet–Parker and tearing mode theory is a consequence of the reconnection layer geometry (equation (2.11)). Because *S*≡*Lv*_*A*_/*η*, decreasing the length of the current sheet *L* or increasing the opening angle of the magnetic X-point $\tan^{-1}\left(B_{y}/B_{x}\right)$ in figure 1 increases the reconnection rate.

In this section, we discuss the factors which control current sheet formation and geometry, and how they affect the rate of MHD reconnection. In §4a, we discuss situations in which ideal MHD theory predicts that the current becomes singular. Although singularity formation is prevented by resistive or kinetic effects in a real plasma, it is reasonable to assume that, if ideal MHD predicts a singularity at a certain location, the current will become large and reconnection is likely to occur there. We will see, however, that it is not necessarily fast reconnection. In §4b, we discuss some of the important modifications to reconnection and singularity formation that appear when the magnetic field lines are tied to a conducting boundary, as they approximately are in the coronae of stars and hot accretion discs. In §4c, we review the fast MHD reconnection model of Petschek and discuss why the model falls short. In §4d, we discuss how microinstabilities could lead to anomalous resistivity that enhances the reconnection rate, but argue that there is no conclusive evidence that they do. In §4e, we go beyond microinstabilities to the broader topic of how turbulence affects reconnection and *vice versa*. In §4f, we discuss an instability known as the plasmoid instability, which breaks up Sweet–Parker current sheets, self-consistently generates turbulence and appears to lead to fast MHD reconnection. Because Sweet–Parker theory predicts that shorter current sheets are thinner, the plasmoid instability may trigger a transition to fast collisionless reconnection by driving the current sheet thickness below the critical value at which collisionless effects become important (§5).

### (a) Formation of current singularities

An example of current singularity formation in an ideal plasma is given in [33], which analyses finite amplitude effects in the ideal internal kink instabilty of a helical magnetic field in cylindrical geometry. This is an *m*=1 perturbation in which the plasma inside the rational surface *r*_s_ defined by *mB*_*ϕ*_(*r*_s_)/*r*_s_+*k*_*z*_*B*_*z*_(*r*_s_)=0 is radially displaced. When finite amplitude effects are considered, the abrupt truncation of the displacement at the rational surface creates a current singularity which reconnects the field. This result was generalized and extended by [34]. In these cases, reconnection is initiated on the dynamical, or Alfvén time scale of the plasma and is fast, even within the resistive MHD model.

**Figure 2.**
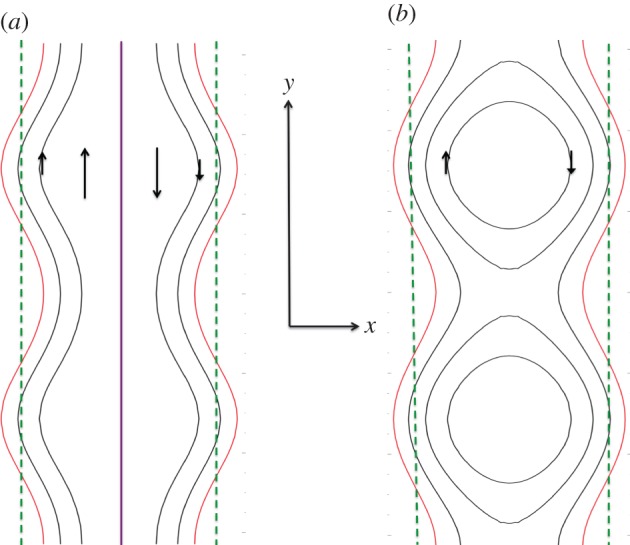
Magnetic field geometries for the Taylor–Hahm–Kulsrud problem. The dotted green lines are the original boundaries and the red curves are the new boundaries. (*a*) The field has the same topology as the original field, but a current singularity (purple line) at the mid-plane *x*=0. (*b*) The field has a reconnected topology but no current sheet.

Singularities can also form in stable plasmas, but they must be driven. The so-called Taylor problem shown in figure 2 (JB Taylor 1982, private communication; [35], also known as the Taylor–Hahm–Kulsrud problem) is an example. A sheared magnetic field $\hat{z}B_{T}+\hat{y}B_{0}x/a$ with *B*_0_/*B*_T_≪1 is bounded by perfectly conducting plates at *x*=±*a*. The system is in equilibrium and stable to tearing. The boundaries are then sinusoidally perturbed slowly enough that the system remains in equilibrium everywhere except near the neutral line (the Alfvén transit time from the boundary to point *x* diverges logarithmically as x→0).

To first order in the perturbation amplitude, two equilibria are compatible with the perturbed system. One (sketched in figure 2*a*) has the same topology as the original field, but has a current sheet at *x*=0.^4^ The other (sketched in figure 2*b*) has different topology: a chain of magnetic islands. The magnetic energy of the current sheet equilibrium exceeds that of the island equilibrium.

Initially, the system approaches the current sheet equilibrium, with a peak current that grows as *t* in a layer that narrows as *t*^−1^. The resistive term in equation (2.4) becomes important at time $t∼{\left(a^{2}\tau_{A}^{2}/\eta \right)}^{1/3}$ after which the system reconnects at a rate proportional to *S*^−3/5^, the same *S* dependence as the slab-tearing mode growth rate. That is, reconnection sets in slowly, and, when it does, it is a slow reconnection. Alternative reconnection scenarios for the Taylor–Hahm–Kulsrud problem are reviewed in [37]. It is found that fast reconnection occurs if the boundary perturbation amplitude is large enough that the current sheet breaks up into plasmoids, due to an instability discussed in §4f. However, the time for reconnection onset is set by the Alfvén travel time from the boundary towards the mid-plane, which due to the reversal of the sheared field is long.

The results in [33–35] suggest that, when current sheets are formed by a dynamical instability, MHD reconnection is fast, but without dynamical instability (or a supervening process like the plasmoid instability) MHD reconnection is slow.

### (b) Reconnection in line-tied magnetic fields

If an ideal plasma is bounded by a perfect conductor, the magnetic flux through the boundary is fixed. This condition is sometimes used to model a magnetically confined plasma surrounded by a conducting wall. It is also used to describe the tenuous coronae of the Sun, other stars, and accretion discs which overlay highly conducting, denser plasmas. In the astrophysical cases, the underlying plasma is in motion due to its own internal dynamics and drags the field lines with it while preserving magnetic flux.

In general, line-tied plasmas do not have rational surfaces in the sense described in §2. For example, the helically symmetric perturbations which can satisfy the rational surface condition *mB*_*ϕ*_/*r*_s_+*k*_*z*_*B*_*z*_(*r*_s_)=0 at *r*=*r*_s_ are incompatible with boundary conditions at constant *z*. Resistive instabilities in line-tied systems were investigated in [38,39], in which it was shown that the resistive tearing mode growth rate holds if a plasma is so long that the tearing layers of adjacent modes in a periodic plasma, the spacing of which is inversely proportional to length, overlap. However, in shorter plasmas, perturbations grow on the resistive diffusion time scale or not at all. As even the MHD tearing mode growth rates are very slow in astrophysical coronae due to the large values of *S*, some other cause must be found for fast reconnection in line-tied plasmas.

A mechanism for fast MHD reconnection in line-tied systems through singularity formation was proposed in seminal papers by Syrovatskii [40] and Parker [41]. In [40], Syrovatskii considered boundary-driven evolution of 2D, line-tied, current-free magnetic fields and showed that boundary perturbations of the field generally deform null points into singular current sheets which could be sites of energy release through rapid reconnection, resulting in solar flares.

Syrovatskii’s theory has been extended to three dimensions [42,43]. These works highlight the key role of magnetic separator surfaces, surfaces across which the field line connectivity changes discontinuously. The role of separator surfaces as likely sites for current sheet formation and reconnection has been further generalized to systems that lack null points through the introduction of quasi-separatrix layers (QSLs) [44–46]. QSLs have been identified in the LAPD experiment and in kinetic simulations of line-tied reconnection [47]. To the extent that QSLs can be observationally identified, they may be useful for predicting the onset of solar flares.

Note that QSLs themselves are not necessarily reconnecting. As we discussed below equation (2.2), in MHD there is a well-defined field line velocity everywhere except at null points or on closed field lines. But as the following example illustrates small perturbations can create current sheets at QSLs which probably dissipate by reconnection.

**Figure 3.**
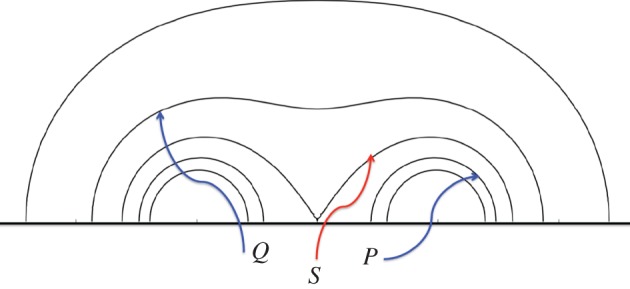
Magnetic field with a separatrix (*S*). The field lines labelled *P* and *Q* are inside and outside the separatrix, respectively. If *P* and *Q* are very close to *S*, then *Q* is nearly twice as long as *P*. The thick black horizontal line represents a boundary to which the field lines are tied. Shearing motions of the boundary perpendicular to the plane of the figure (*y* direction) will produce a current sheet unless the shear Δ*y* of *Q* is twice the shear of *P*.

Figure 3 shows a current-free, line-tied magnetic field with a discontinuity in connectivity. If the field is sheared by motions on the bottom boundary, a current singularity develops along the separatrix [48,49] . This occurs because equilibrium requires constant magnetic shear per unit length, while the total shear must give the correct footpoint displacement. This leads to discontinuities in the magnitude of the sheared field in situations where the equilibrium field lines discontinuously change their length.

The mathematical basis for the argument is as follows. For simplicity, consider a force-free magnetic field of the form $\text{B}=\hat{y}\times \nabla \psi (x,z)+\hat{y}B_{y}(x,z)$; the flux function *ψ* is constant on field lines. Force balance in *y* requires *B*_*y*_=*B*_*y*_(*ψ*); *B*_*y*_ is also constant on field lines. Let the shear of a field line from one footpoint to another be Δ*y*(*ψ*). Integrating the field line equation *dy*/*ds*=*B*_*y*_/|**∇***ψ*| from one endpoint to another along a line of constant *ψ* gives

4.1 $$\Delta y(\psi )=B_{y}(\psi )\int \frac{ds}{\left|\nabla \psi \right|}.$$

Consider the two field lines labelled *P* and *Q* just inside and just outside the separatrix in figure 3. The integral over *Q* is twice the integral over *P*. Therefore, unless Δ*y*(*ψ*_*Q*_)=2Δ*y*(*ψ*_*P*_), *B*_*y*_ must have a jump across the separatrix; *B*_*y*_(*ψ*_*Q*_)≠*B*_*y*_(*ψ*_*P*_). In this sense, a separatrix is an accident waiting to happen. It is reasonable to suppose that the current sheet forms on the fast Alfvén time scale because shear propagates along field lines at the Alfvén speed.

**Figure 4.**
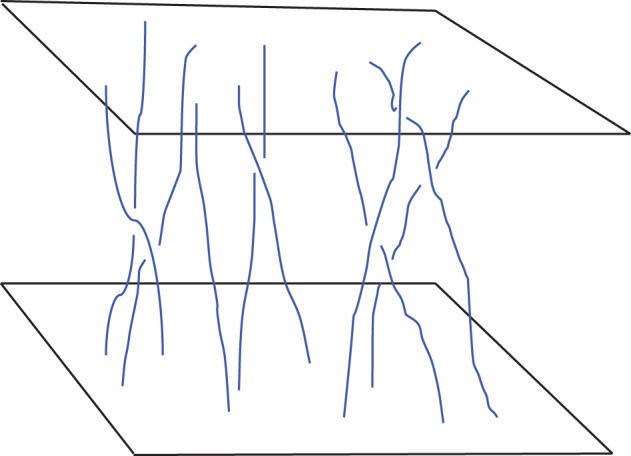
Set-up for the magnetostatic equilibrium problem posed by Parker [41]. Magnetic field lines are anchored to perfectly conducting plates which twist, shear and braid the field through horizontal motions.

Parker [41] addressed situations with the simpler magnetic topology shown in figure 4. Two perfectly conducting surfaces, representing independently moving portions of the photosphere, are connected by an initially uniform magnetic field. If the footpoints of the field lines are displaced slowly by random motions on the plates, the field will evolve through a series of equilibria and, in the ideal limit, will preserve the mapping between footpoints. Parker conjectured that, in general, smooth equilibria are impossible under this type of boundary driving. The field will remain force free almost everywhere, but current singularities will develop. The energy released by reconnection of the current sheets could both heat the corona (many small current sheets per volume, each carrying little energy) and power flares (comparatively rare events, large stored energy).

Parker’s claim has not yet been proved in general, although it has attracted wide attention and is the subject of too many papers to cite here (see [50], for a recent review). However, whether actual singularities are formed or not, many numerical experiments have shown that even very simple footpoint motions will, over time, induce sheets and filaments of high current density in which the dissipation is large, e.g. [51–54].

Rapid energy release in such simulations often comes in the form of large bursts, suggesting instability. This is supported by a theoretical argument. It was shown by construction in [55] that the small amplitude limit of the Parker problem, in which the field lines are nearly straight and vertical, has a unique, smooth solution. Therefore, suppose the field has become nonlinearly distorted by shearing and twisting while retaining the property that all field lines pass from one plate to the other. These distortions increase the free energy of the field and may drive it to the point that it becomes unstable. At the instability threshold there is a perturbation with zero frequency. That is, there are two slightly different equilibrium states accessible to the system. It was shown in [56] that at most one of these states can be smooth; the other has current singularities. This identifies current sheet formation with MHD instability and connects singularity formation in line-tied plasmas to the slow and fast modes of singularity formation discussed in §4a.

Similar behaviour was found for simulations of the Parker problem that included resistivity, and therefore did not fix topology [57]. Two regimes were found. In one, although the footpoint connectivity changed rapidly, the evolution of the field was diffusive. In the second case, which had slightly higher initial magnetic energy, there was clearly a dynamical phase that was interpreted as reconnection triggered by an instability.

These numerical results are suggestive but still incomplete. Because *S* is so large in the solar corona, it is important to determine whether the equilibrium of line-tied magnetic fields has true current singularities or merely very large and intermittent currents, to characterize the statistical properties of the sheets and to determine how the equilibrium level and spatial and temporal intermittency of energy release depend on *S* [58]. This is necessary for testing the theory with observations of coronal dynamics and energetics.

### (c) Petschek reconnection model

By the early 1960s, a serious discrepancy was recognized between the measured magnetic reconnection rates and those predicted by the Sweet–Parker model, which is hampered by the limited mass flow through the very narrow current channel of constant width, as shown in figure 1. To remove this major hurdle of the Sweet–Parker theory, Petschek proposed that introduction of wedge-shaped shocks, with a sudden jump of magnetic field direction, would greatly speed up the mass flow in the outflow region [59]. In the Petschek model the resistive layer is short, and most of the fluid is deflected around it by the two pairs of slow mode magnetosonic shocks. Reconnection rates *v*_in_ of order $v_{A}/\ln S$ can be realized according to this model.

Two-dimensional numerical simulations of MHD reconnection with a local, current-dependent enhancement of the magnetic diffusivity *η* spontaneously reproduced the Petschek reconnection profile and fast reconnection rate [60,61]. In later resistive MHD simulations, the Petschek slow shock configuration was found to be sustained *only* if enhanced resistivity was imposed within the high current density region [62]. The Petschek slow shock has not been conclusively identified either in the laboratory or in space plasmas and has fallen out of favour for these reasons and because it appears to be unstable [63,64]. So far, no persuasive theory has been developed to physically justify this model. Kinetic processes which could locally enhance *η* are discussed in following subsection.

### (d) Anomalous resistivity

If the drag on electrons due to Coulomb collisions were supplemented by scattering from small-scale waves or fluctuations, *η* could be enhanced in the diffusion region. The waves could be produced by the strong currents and gradients in the reconnection layer, which can excite microinstabilities. Electromagnetic fluctuations consistent with such instabilities and correlated with enhanced reconnection rates have been detected in laboratory reconnection layers [65]. Whether the fluctuations actually *cause* faster reconnection is still an open question. To date, no microinstability accompanied by a clear physical mechanism which could be responsible for anomalous resistivity has been identified [4].

If anomalous resistivity does exist, it might limit the electron drift *u*_e_=−*J*/*en*_e_ to some marginally stable value *u*_ec_. From Ampere’s law, we can approximate *u*_e_ in a layer of width *δ* as

4.2 $$u_{e}∼v_{A}\frac{d_{i}}{\delta },$$

where *d*_i_ is the ion skin depth.^5^ Using equations (2.11) and (4.2), we can write the reconnection rate as

4.3 $$\frac{v_{\mathrm{in}}}{v_{A}}∼\frac{v_{A}}{u_{\mathrm{ec}}}\frac{d_{i}}{L}.$$

In a hydrogen plasma $d_{i}∼2.3\times 10^{7}/n_{i}^{1/2}\;\mathrm{cm}$, generally much less than *L* for global scale current sheets. Equation (4.3) shows that fast reconnection, therefore, requires a very low instability threshold, *u*_ec_/*v*_A_≪1. However, a reasonable value of *u*_ec_ is closer to the ion thermal speed *v*_i_, which is close to *v*_A_ in the reconnection layer. It follows that, if *u*_ec_=*v*_A_, the layer width *δ* is just *d*_i_. As we will see in §5, this is the critical width at which the two-fluid or kinetic description of reconnection must replace the MHD description.

### (e) Turbulent current sheets and current sheets in turbulence

In numerical simulations of MHD turbulence, the distribution of current becomes highly intermittent [66,67]. Not all of the current layers contain magnetic X-points, and, therefore, not all can be reconnecting. However, the resistive dissipation rate per unit volume within current sheets is much larger than in the plasma as a whole, so they play an outsized role in turbulent energy balance and the nature of the turbulent cascade. These current sheets span a range of sizes, up to the largest turbulent driving scale.

The effect of small-scale turbulence on reconnection of a background field with a large-scale reversal was considered by [68] and later by [69,70]. The former authors performed 2D computations of reconnection in turbulence seeded by initial perturbations. They concluded that the range of Rm that could be probed with the computational resources available was insufficient to quantify the effect of turbulence on the reconnection rate, but suggested that it might be possible to describe the effect of turbulence by an effective resistivity. The latter authors concentrated on the reconnection rate. Their three-dimensional (3D) computations showed the Sweet–Parker reconnection in the absence of turbulence, but faster reconnection, at a rate independent of Rm over the small range of Rm tested, if turbulence was driven in the reconnection layer. They ascribed the enhanced reconnection rate to the presence of multiple X-points and to turbulent field line wandering, which allows the smaller aspect ratio geometry associated with fast reconnection (equation (2.11)) to prevail at individual X-points. Whether it is possible to identify an effective resistivity for reconnection in a turbulent medium remains unclear.

An even greater role for field line wandering is embodied in the proposal that magnetic fields are not frozen in at all in the presence of turbulence [71]. In turbulent flow, neighbouring fluid elements separate rapidly with time. The elements of the deformation matrix **D** defined in equation (2.7), therefore, become very large, or, in the ideal limit, undefined. From equation (2.6) we see that, if the concept of ‘following a fluid element’ ceases to be meaningful, the concept of a magnetic field frozen to fluid elements also breaks down. This breakdown of the frozen field condition is called ‘spontaneous stochasticity’ [72]. As a field that is not frozen in cannot store energy or be amplified by a flow, this behaviour would have drastic consequences if it were universal, which is contradicted by evidence for magnetically powered flares, astrophysical dynamos, etc.

The Cauchy solution for magnetic field evolution takes the fluid motion as given and does not address the question of how that motion is produced or how the magnetic field modifies it. This issue is addressed (in a different context) in [73], in which it is found that the back reaction of the field on a chaotic flow reduces the Lyapunov exponents, i.e. the rate at which neighbouring fluid elements move apart. This is due to the locally large **J**×**B** force that develops as the field lines shear, and is illustrated by explicit solutions for the transient amplification and rapid dissipation of magnetic fields and currents by flow stagnation points [74]. These effects may limit the effectiveness of spontaneous stochasticity.

### (f) Fast magnetohydrodynamic reconnection mediated by the plasmoid instability

In §3, we discussed how *L* is influenced by global geometry. It can also be determined locally, by instability. Recently, a theory for fast reconnection of MHD current sheets was developed [75,76], based on the discovery that the Sweet–Parker reconnection layer is unstable in two dimensions to tearing if the Lundquist number *S* exceeds a threshold of order 10^4^ [77]. Because the instability is driven by the gradient of current, the instability growth rate increases with *S*, scaling as *S*^1/4^ for *S* far above threshold. The wavenumber $k_{\max}$ of the fastest growing mode is of order *L*^−1^*S*^3/8^ and the maximum growth rate $\gamma_{\max}$ is of order *S*^1/4^*v*_A_/*L*. The nonlinear structures formed by the instability are called plasmoids, and the instability is now referred to as the plasmoid instability.

The outcome of the instability, which has been studied in two and three dimensions, with and without a perpendicular or guide field, and with a variety of plasma models (resistive MHD, two fluid and full kinetic; see §5b) appears to be a broad, turbulent, highly time-dependent reconnection layer filled with magnetically detached ‘plasmoids’—flux ropes or eddies, depending on the presence or absence of a guide field. This is so even though the mechanisms for producing the plasmoids may differ from model to model. The relationship between current sheet aspect ratio and reconnection rate (equation (2.8)) implies that the threshold *S* for instability corresponds to *δ*/*L*∼*v*_in_/*v*_A_∼10^−2^. This is roughly what is found in MHD simulations [78–80]. In both two and three dimensions, the turbulent layer with its multitude of magnetic islands is a favourable environment for non-thermal particle acceleration by a stochastic Fermi mechanism [81]. We discuss the two-fluid and kinetic counterpart of these results in §5b.

Plasmoid instability-driven turbulence appears self-consistently in layers that have achieved the critical aspect ratio. This is in contrast with turbulence which is mechanically forced in the reconnection layer, as in [69,70]. The plasmoid instability appears to offer a viable path to fast reconnection once a sufficiently thin current sheet has formed.

## 5. Two-fluid analysis of the magnetic reconnection layer

In the one-fluid MHD formulation, the difference between the fluid velocities of electrons and ions is supposed to be much smaller than the Alfvén velocity or the ion velocity. This one-fluid approximation criterion can break down in the reconnection layer because of the sizable amount of neutral sheet current flowing there; namely, electrons and ions move quite differently and the ‘two-fluid’ formulation becomes more appropriate. Here, by two-fluid formulation, we mean that electrons and ions are treated as separate fluids and the reconnection dynamics can be described by the generalized Ohm’s law, assuming quite different velocity distributions of electrons and ions [82,83]. Generally, the two-fluid formulation implies that the electron and ion distribution functions are close to shifted Maxwellians, and this imposes constraints on the level of collisionality required for such description.

We saw from equation (4.2) that the drift is of order *v*_A_ when the layer width *δ* is of order *d*_i_. This regime is dubbed collisionless reconnection because the condition *δ*≤*d*_i_ can be expressed in terms of the collisional mean free path *l*_*c*_ as *l*_*c*_>(*m*_*e*_/*m*_i_)^1/2^*L*.^6^

The condition *δ*/*d*_i_<1 can be written quantitatively for the Sweet–Parker scaling [83] as

5.1 $$L<\omega_{\mathrm{ce}}\tau_{e}d_{i}.=\frac{1.1\times 10^{13}}{\left(\lambda /10\right)}{\left(\frac{T_{e}}{n_{e}}\right)}^{3/2}B,$$

where λ, the Coulomb logarithm, is typically 10–30, and *L*, *B*, *T*_e_ and *n*_e_ are in units of cm, G, K and cm^−3^, respectively. The condition *δ*/*r*_i_<1, (strong guide field case) is, assuming *T*_e_=*T*_i_,

5.2 $$L<\frac{2.0\times 10^{-2}}{\left(\lambda /10\right)}\frac{T_{e}^{5/2}}{n_{e}^{1/2}B}.$$

Inserting typical parameters shows that collisionless effects are important in space plasmas and many laboratory plasmas, that the solar corona is close to marginal and that reconnection in the interstellar medium is generally collisional unless current sheets form on scales many orders of magnitude below global length scales.

In the two-fluid formulation, the Ohm’s law of MHD (equation (2.3)) should be replaced by the generalized Ohm’s law, which is the momentum equation for the electron fluid

5.3 $$\text{E}+\frac{\text{u}}{c}\times \text{B}=\frac{\text{J}}{\sigma }+\frac{\text{J}\times \text{B}}{en_{e}c}-\frac{\nabla \cdot {\text{P}}_{e}}{en_{e}}-\frac{m_{e}}{e}\frac{d{\text{u}}_{e}}{dt}.$$

Here, the conventional notation is used with subscript ‘e’ denoting electrons and **P**_e_ the electron pressure tensor. In equation (5.3), the first term on the r.h.s. is negligible in collisionless reconnection, the second term represents the Hall term. Equation (5.3) can be reduced to the MHD Ohm’s law by setting **u**_e_=**u**_i_=**u**, and by neglecting the electron inertia and pressure tensor terms.

**Figure 5.**
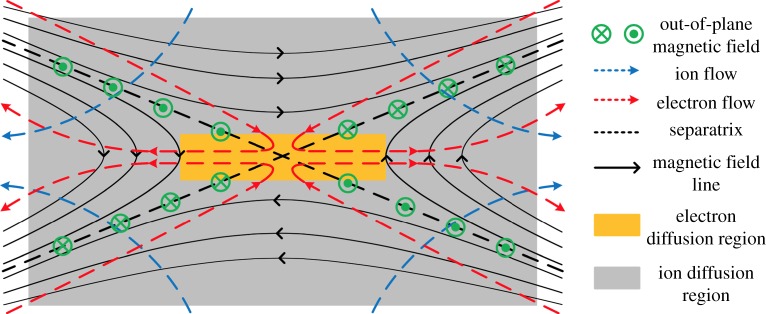
Schematic of two-fluid reconnection. Ions decouple from electrons in the ion diffusion region (grey colour). Electrons are frozen to the field lines until they reach the electron diffusion region (orange colour). The electron flow pattern creates a quadrupole out-of-plane magnetic field, a signature of the Hall effect.

Two-fluid reconnection is shown in figure 5 [84]. In the grey region, ions are demagnetized but electrons are still magnetized and the relative drift velocity between electrons and ions can be large. Differential motion between the magnetized electrons and the unmagnetized ions generates strong Hall currents in the reconnection layer, as shown by the red broken lines in figure 5. Most of the region shown in grey, where ions are demagnetized, is called the ‘ion diffusion region’ with *c***E**+**u**_i_×**B**≠0. Electrons are still magnetized (*c***E**+**u**_e_×**B**=0) until they approach the X-point. This central region near the X-point is called the ‘electron diffusion region’. The inertia term and pressure tensor term become large relative to the other terms in the electron diffusion region. Generally in equation (5.3), plasma parameters (*n*_e_ and all vectors) should include fluctuating components and *σ* denotes the classical Spitzer resistivity based on Coulomb collisions (i.e. fluctuations are treated explicitly, not as a resistivity).

A large out-of-plane electric field is generated by the Hall currents at the reconnection layer (**J**_Hall_×**B**) and increases the reconnection rate by inducing rapid movement of the reconnecting field lines [4,85]

5.4 $$|{\text{u}}_{e}\times {\text{B}}_{\mathrm{rec}}|\approx cE_{\mathrm{rec}},$$

where **B**_rec_ is the reconnecting magnetic field component and *E*_rec_ is the reconnection electric field. The dynamics of electrons and ions in collisionless reconnection was analytically discussed first by Sonnerup [86] and later by Uzdensky & Kulsrud [87] more exactly. In this ion diffusion region, the magnetized electron flows and demagnetized ion flows are decoupled and the electron fluid inflow and outflow towards the exhaust are dominant; **u**_e_=−**J**_e_/*en*_e_. From Ampere’s law, this creates an out-of-plane magnetic field with quadrupolar structure. Thus there are two distinctive features in two-fluid reconnection: (i) an out-of-plane quadrupole Hall magnetic field and (ii) a fast reconnection rate caused by the large out-of-plane (Hall) electric field [4,88]. This picture is supported experimentally. In studies of the local two-fluid physics of the reconnection layer, Hall effects were observed in MRX [85,89], MST [90] and SSX [91].

Another important feature of the two-fluid reconnection layer is that the shape of the reconnection layer is similar to that of the Petschek model, namely the outflow channel is expanding, thus not hampering plasma outflow as is the case for Sweet–Parker model. This is an important characteristic for accommodating fast reconnection. However, the magnetosonic slow mode shocks predicted by Petschek’s theory are absent in two-fluid reconnection. It should be also noted that the Hall fields and the enhanced reconnection rate are seen even with the presence of significant collisions, namely when the mean free path of electrons is comparable with the thickness of the reconnection layer [4,89]. It should be noted, furthermore, that in recent simulations of electron–positron pair plasmas [92,93], in which the opposite motions of positrons and electrons cancel the Hall effect, fast reconnection is still observed. Thus, the question remains as to whether a kinetic effect other than the Hall effect plays a key role in fast reconnection in kinetic plasma.

### (a) Experimental study of the dynamics of the two-fluid diffusion layer and identification of a two-scale diffusion region

**Figure 6.**
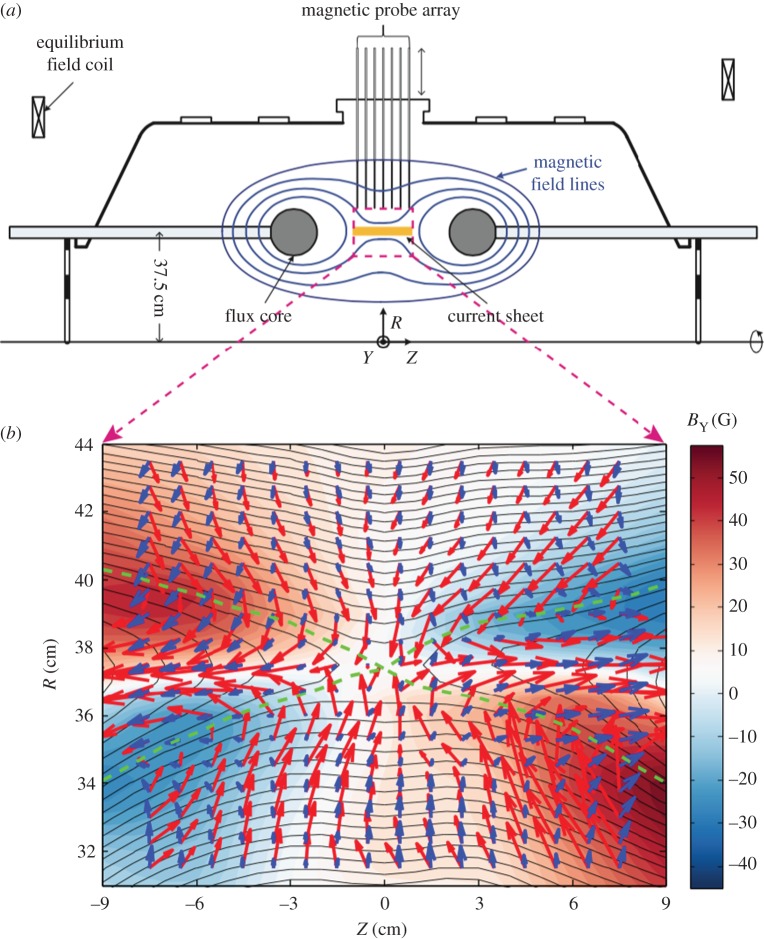
(*a*) MRX apparatus and reconnection drive. (*b*) Measured flow vectors (length represents velocity) of electrons (red arrows) and ions (blue) in the full reconnection plane together with poloidal flux contours (which represent reconnecting field line components projected in the reconnection plane) and out-of-plane field contours; 1 cm vector length stands for 2×10^6^ cm s^−1^, colour contours represent out-of-plane field strength and green broken lines depict (experimentally identified) separatrix lines. Toroidal symmetry is assumed.

Using extensive diagnostics, the dynamics of plasma particles and mechanisms for energy conversion in the reconnection layer were recently documented in MRX, as shown in figure 6*a*. The main diagnostic is a 2D magnetic probe array that measures the evolution of all three components of the magnetic field at more than 200 locations in the reconnection plane [9,84], using miniature pick-up coils with resolution as small as a few electron skin depths (2–6 mm). The local ion temperature is measured by the ion dynamics spectroscopy probe (IDSP) [94]. The ion flow vectors are measured by Mach probes. Triple Langmuir probes are used to measure electron temperature and density. The electric field in the reconnection plane is deduced from the in-plane potential profile measured by a floating potential probe and Langmuir probes. The out-of-plane reconnection field is primarily inductive, and can be measured by following movements of the reconnecting flux lines.

In the MRX experiment, a two-scale diffusion layer was identified in which the electron diffusion layer resides inside the ion diffusion layer, the width of which is the ion skin depth *d*_i_ [4,95,96]. Here, we define the ion diffusion layer as the regime of *c***E**+**u**_i_×**B**≠0 and the electron diffusion layer as the regime of *c***E**+**u**_e_×**B**≠0, as defined in figure 4. The ion diffusion layer has roughly a width of the ion skin depth (*c*/*ω*_pi_), about 5–6 cm in this experiment, and the electron diffusion region width is 5–10 times the electron skin depth (*d*_e_≡*c*/*ω*_pe_≈1 mm).

Furthermore, it was found that demagnetized electrons are accelerated along the outflow direction and within the reconnection plane. The width of the electron outflow was shown to scale with the electron skin depth as 8 *d*_e_, which is three to five times wider than predicted by 2D numerical simulations [95,96]. While the electron outflow seems to slow down due to dissipation in the electron diffusion region, the total electron outflow flux remains independent of the width of the electron diffusion region. We note that, despite the presence of the narrow electron diffusion region, the reconnection rate is still primarily determined by the Hall electric field [97]. This is in accord with predictions from the Geospace Environmental Modeling challenge [98], a coordinated set of numerical simulations of a tearing unstable current sheet using a variety of fluid and plasma models.

**Figure 7.**
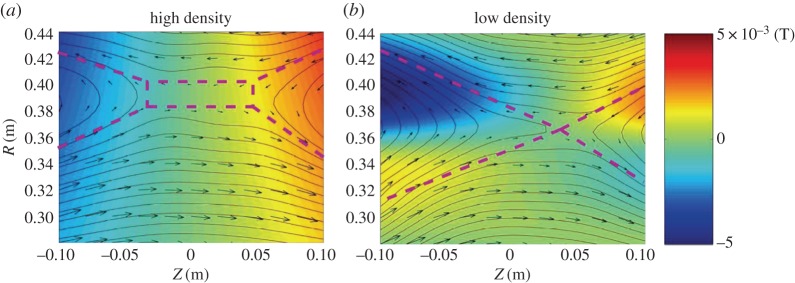
Comparison of the experimentally measured reconnection layer profile for two cases: (*a*) collisional regime (λ_mfp_≪*δ*_sheath_) and (*b*) nearly collisionless regime (λ_mfp_>*δ*_sheath_). The in-plane magnetic field is shown as arrows and the out-of plane field component shown by the colour codes ranged from −50 G to 50 G. Dashed pink lines show that the magnetic configuration changes from an elongated current sheet (Sweet–Parker type in (*a*)) to a double-wedge shape (Petschek-like) as collisionality is reduced. The predicted quadrupole structure of the out-of-plane magnetic component, a signature of Hall effects, is observed in (*b*). (Adapted from [85].)

In MRX, the measured profile of the neutral sheet changes drastically from the high-density (collisional) to low-density (nearly collisionless) cases. In the high plasma density case, shown in figure 7*a*, where the mean free path is much shorter than the sheet thickness, a rectangular-shaped neutral sheet profile characteristic of the Sweet–Parker model is seen together with the observed classical reconnection rate. There is no recognizable out-of-plane Hall field in this case. In the case of low plasma density, shown in figure 7*b*, where the electron mean free path is larger than the sheet thickness, the Hall MHD effects become dominant as indicated by the out-of-plane field depicted by the colour code. A double-wedge-shaped sheet profile of Petschek type, which is shown in the flux contours of the reconnecting field in figure 7*b*, is significantly different from that of the Sweet–Parker model (figure 7*a*), and a fast reconnection rate is measured. The observed fast reconnection is also consistent with the expanding shape of the outflow region just as in the Petschek model. However, a slow shock, an important signature of the Petschek model, has not been identified to date even in this collisionless regime.

### (b) Multiple reconnection layers in large collisionless systems

Laboratory fusion plasmas and astrophysical systems are generally much larger than the key microphysical scales such as the ion skin depth and ion gyroradius. Most of the work on reconnection in the past, both numerical and experimental, has investigated relatively small systems—10–100 ion skin depths. In §4f, we discussed the resistive MHD instability of large aspect ratio current sheets that breaks them up into multiple plasmoids. Similar behaviour is theoretically predicted [99] and seen in two-fluid and kinetic simulations of much larger systems—up to 10^4−5^ ion kinetic scales that have recently become feasible due to increased high-performance computing capabilities [100,101].

**Figure 8.**
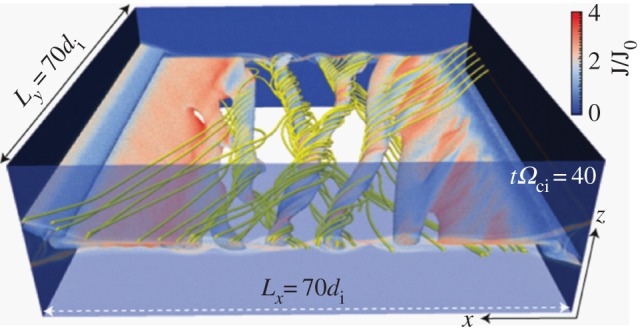
Formation of primary flux ropes observed by a 3D simulation: [102]. At early time, the tearing instability forms flux ropes as illustrated by an iso-surface of the particle density coloured by the magnitude of the current density (normalized) along with sample magnetic field lines (yellow). Note that their coordinate system (*Z*, *x*, *y*) should correspond to the MRX (*R*, *Z*, *y*) system.

In [100], it is found from 2D calculations that a collisionless reconnection layer breaks up into many islands and current layers, generating a highly turbulent reconnection region. Recent simulation studies have been extended to three dimensions. In a 3D simulation with guide field, the plasmoid instability leads to the generic formation of multiple flux ropes [102], generating a highly turbulent reconnection region, as shown in figure 8. The majority of the flux ropes are formed by secondary instabilities within the electron layers. These flux ropes appear spontaneously, leading to a turbulent reconnection layer that significantly broadens the electron and ion diffusion regions. New flux ropes spontaneously appear within these layers, leading to a turbulent evolution where electron physics plays a central role. We expect quite impulsive reconnection rates in this situation. New approaches are required to properly describe this turbulent layer.

An example of flux rope formation was shown in the MRX experiment [103] in which an imbalance of incoming flux and outgoing flux at the electron diffusion region generates flux ropes and the reconnection rate becomes unsteady and fluctuates with large amplitude. Another study of the collisionless plasmoid instability has also recently been reported in [104]; see also [105].

These new advances provide near-universal mechanisms to directly couple local kinetic-scale physics to global MHD-scale physics based on multiple X-lines in all regimes of collisionality. However, despite great progress on both the experimental and theoretical fronts, most of the natural space and astrophysical systems motivating reconnection research have a much larger separation of scales between global system size *L* and the plasma microscopic scales (e.g. in the solar corona, *L*∼10^5^ km, whereas the ion gyroradius could be approx. 1 m). This huge separation of scales exemplifies the scaling problem of reconnection research: how does one extrapolate the knowledge gained from studying relatively small and intermediate-size systems, both laboratory and numerical, to the real world?

At the moment, there are plans to experimentally study magnetic reconnection in larger accessible regimes in the ‘phase diagram’ of reconnection, using improved diagnostics and state-of-the-art computing tools [106]. This will enable us to make improved predictions regarding space and astrophysical plasmas. Understanding the generation and influence of secondary reconnection instabilities is one of the primary goals of two new reconnection experiments, TREX [107] and FLARE, a multi-institutional collaborative experiment currently under construction at the Princeton Plasma Physics Laboratory [108]. Accordingly, new kinetic simulation efforts will be directed at modelling these devices in order to validate numerical codes and test theoretical ideas.

## 6. Energy flow and partitioning in a prototypical two-fluid magnetic reconnection layer

As we discussed earlier, one of the most important features of magnetic reconnection is that significant acceleration and heating of plasma particles occurs at the expense of magnetic energy. An example of this efficient energy conversion is the observation of large numbers of high-energy electrons associated with the reconnection of magnetic field lines in solar flares. In the reconnection region of the Earth’s magnetosphere and the solar wind, plasma outflows have been measured *in situ* by satellites. Despite this evidence, the exact quantitative characteristics of bulk plasma heating, particle acceleration and energy flow channels have not been addressed until recently [84,109–111].

In the Sweet–Parker model, based on resistive MHD, the energy dissipation rate is small (∼(*B*^2^/2*μ*_0_)*v*_A_*L*/*S*^1/2^) due to the slow reconnection rate [2,17,112]. It is important to note that the outgoing magnetic energy flux through the thin diffusion region is much smaller than the incoming magnetic energy in this model (figure 1). Almost all of the incoming magnetic energy is expected to be converted to particle energy within the narrow diffusion region (*S*≫1). The plasma is slowly heated by classical resistive dissipation (*ηJ*^2^) in the diffusion region and is accelerated to the Alfvén velocity due to both the pressure gradient and magnetic tension forces. In the exhaust, there is an equipartition between the flow and enthalpy increase, $\Delta (5nk_{B}T/2)∼nmv_{\mathrm{out}}^{2}/2$, indicating that magnetic reconnection generates Alfvénic flows of heated plasma at the end of the very narrow exhaust [2,9]. Recent space observations and numerical simulations show, however, that the situation is different in collisionless reconnection [109,111,113]. The main reason is now considered to be two-fluid physics dominant in the reconnection layer.

It has been recently reported that the energy conversion in a laboratory reconnection layer occurs in a much larger region of the reconnection layer than previously considered [9,84]. This experimental study of the reconnection layer was carried out in the two-fluid regime. The mechanisms for energizing plasma particles in the magnetic reconnection layer were identified, and a quantitative inventory of the energy conversion process was presented for the first time in a well-defined reconnection layer of variable size. We summarize the data below.

### (a) Electron dynamics and heating in the reconnection layer

**Figure 9.**
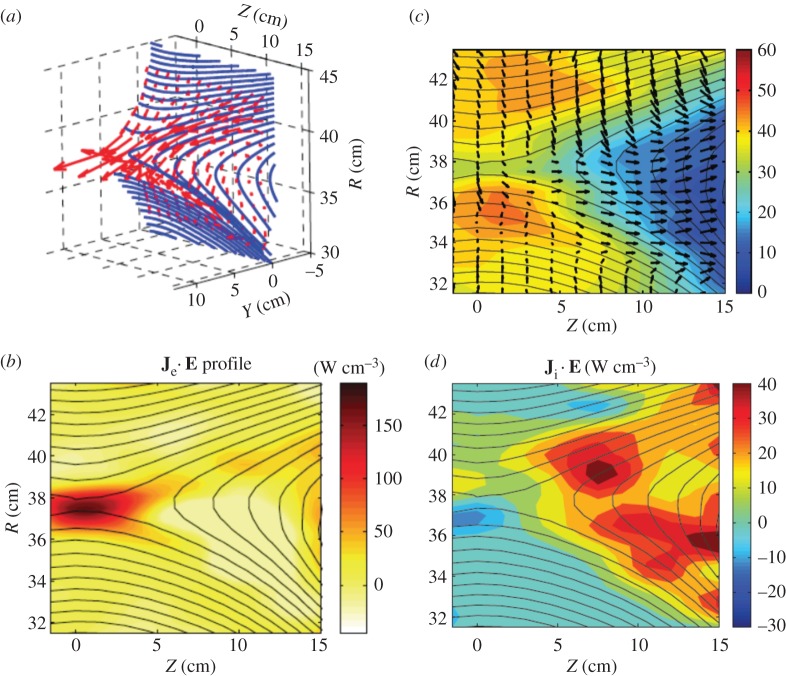
Flow vectors of electrons in 3D views (*a*) and the energy deposition rate to electrons. The high-energy deposition is primarily due to **J**_e_⋅**E**, which is concentrated in the electron diffusion region (*b*). Ion flow vectors in the potential well (in colour contours) (*c*); magnetic field lines shown as flux function contours show that the electric potential is constant along field lines. (*d*) The energy deposition to ions described by **J**_i_⋅**E** occurs across the separatrices, and in a much wider region than for electrons [84,94].

Measured electron flow vectors and field lines in the reconnection half-plane and its perspective view are shown by figure 9*a* in 3D geometry [84]. While ions and electrons move together with the field lines before entering the ion diffusion region, electrons move much faster as they approach the X-point region and the heating term **J**_e_⋅**E** is concentrated near the X-point, as seen in figure 9*b*.

In MRX, the electron flow vectors in the reconnection plane are derived from the electron current profile reconstructed from the magnetic profile measured by fine-scale magnetic probes and the local electron density measured by Langmuir probes. As conjectured by the two-fluid model, field lines and magnetized electrons move together towards the X-point *B*=0 at the centre of the layer. The electrons remain magnetized through the ion diffusion region and flow towards the X-point. Near the electron diffusion region, the magnetic field strength drops significantly, thereby driving up the in-plane electron drift speed (*c*|*E*/*B*|) and ejecting high-velocity electrons into the reconnection outflow exhaust. Simultaneously, the electrons are also accelerated in the out-of-plane direction by the reconnection *E* field. This feature is clearly seen in figure 9*a*. The electron heating occurs in the electron diffusion region and is transported quickly along the magnetic field lines, due to strong parallel heat conduction. Consequently, the electron temperature in the exhaust region is higher than in the inflow region. This observation agrees with recent observations of bulk electron heating in the reconnection exhaust region at the dayside magnetopause [114]. We note that superthermal electrons were not observed in the operation regime of MRX, probably due to the relatively small system size and, to some extent, to collisions of electrons with ions.

### (b) Ion dynamics, acceleration and heating in the reconnection layer

The flow of magnetized electrons, which causes the Hall effect, also produces a strong electric field in the reconnection plane [94]. The field is strongest across the separatrices, which separate incoming field lines from the exhaust of reconnected field lines, as shown in figure 9*c*. It is experimentally verified in MRX that a saddle-shaped electric potential profile is formed in the reconnection plane in order to balance the Lorentz force on the electron flows [94]. A strong in-plane electric field is generated near the separatrices with a wider and deeper potential well downstream. The MRX potential data are consistent with measurements from the CLUSTER spacecraft [115]. The in-plane electric field (or potential gradient) is largely perpendicular to the local magnetic field lines.

The electric potential is seen to be nearly constant along a poloidal flux contour (or magnetic field line), as seen in figure 9*c* in the reconnection half-plane. This figure shows that the large electric field across the separatrices is present in a significantly larger area of the reconnection layer (*L*≫*d*_i_) than the region in which field line breaking and reconnection occur. Electrostatic acceleration of ions is observed near the separatrices due to this strong electric field. In MRX, the spatial scale of the electric field is approximately 2 cm, smaller than the ion gyroradius (approx. 8 cm). This situation induces an electrostatic acceleration of ions through the separatrices. Figure 9*c* shows the 2D profile of ion flow vectors measured by Mach probes, along with contours of magnetic flux. The ion flows change direction at the separatrices and are accelerated in both the *Z* and *R* directions. The energy deposition rate on ions is concentrated near the separatrices in the exhaust region, as seen in figure 9*d*. Notable heating is observed as the ions flow out to the exhaust from the X-region. The cause of this anomalously rapid slowdown of ions, together with ion heating, is considered to be the remagnetization of the exiting ions. Whether the increase in ion temperature is mainly due to deflection by the magnetic field or involves an actual increase of entropy due to interaction with small-scale electromagnetic fluctuations is not yet clear.

### (c) Quantitative study of energy conversion and partitioning in a prototypical reconnection layer

Recently, the first quantitative measurements of the acceleration and heating of both electrons and ions were carried out in the nearly collisionless MRX reconnection layer. It was demonstrated that half of the incoming magnetic energy is converted to particle energy at a remarkably fast rate [110]. It was also found that, within the collisionless reconnection layer, the energy deposited in the ions is more than twice as large as that deposited in the electrons. Furthermore, a non-negligible amount of magnetic energy flows out of the exhaust. It is important to note that the energy deposition rate to electrons (large value of **J**_e_⋅**E**) is highly concentrated near the X-point. The term **J**_e_⋅**E** can be decomposed into **J**_e_⊥⋅**E**_⊥_+*J*_e_∥*E*_∥_, i.e. separating the inner product into that of the perpendicular and parallel components with respect to the local magnetic field lines. Near the X-point where energy deposition is maximum, **J**_e_⊥⋅**E**_⊥_ is larger than *J*_e_∥*E*_∥_ by more than an order of magnitude. Owing to the potential electric field described above, the conversion of magnetic energy occurs across a region significantly larger in area than the narrow electron diffusion region predicted by previous 2D simulations. A saddle-shaped electrostatic potential profile is experimentally verified within the reconnection plane in both the experiment and simulations, and as a result ions are accelerated by the resulting electric field at the separatrices [94]. This acceleration and heating of ions happens in a wide region extending over an ion skin depth—the so-called ion diffusion region. When the energy deposition rate to ions, **J**_i_⋅**E**, is directionally decomposed, the perpendicular component, **J**_i_⊥⋅**E**_⊥_, is again found to be dominant over *J*_i_∥*E*_∥_ in the regions where energy deposition to ions is maximum [9,84].

Recently, key aspects of the structure and energetics of the reconnection layer studied in MRX were identified in space plasmas by the magnetospheric multi-scale mission (MMS) [116]. It is gratifying to see such commonality between laboratory and space plasmas. The MMS results also demonstrate the utility of combining experiments, numerical simulations and observations of space plasmas to understand basic physical processes.

These findings with respect to the energy conversion give us the following new perspectives on the reconnection layer in the two-fluid plasmas.

(1) The energy deposition mechanisms for electrons and ions are quite different in the two-fluid reconnection layer. But the energy deposition rates to electrons and ions are dominated by **J**_⊥_⋅**E**_⊥_ (figure 9*a*) for both species (figure 9*a*,*d*) although the electric fields are quite different in the energy deposition regions.
(2) The energy conversion occurs in a wider region of the reconnection layer than the field line breaking region. Based on these results, it would be more appropriate to call this extended reconnection layer the **energy conversion region** rather than the **diffusion region**.
(3) A quantitative inventory of the converted energy concluded that about 50% of the inflowing magnetic energy is converted to particle energy, roughly two-thirds of which is ultimately transferred to ions and one-third to electrons. The other half of the inflowing magnetic energy flows out to the outflow region. The results are consistent with recent space observations. These features of energy conversion and partitioning do not strongly depend on the size of the analysis region over the tested range of scales, approximately two to eight ion skin depths.

The collisionless reconnection picture is to be contrasted with the MHD Sweet–Parker reconnection picture, in which roughly half the incoming magnetic energy is dissipated through Ohmic heating and the remainder to acceleration of the Alfvénic exhaust jet, but with extremely slow rate.

## 7. Summary and discussion

In the Introduction to this paper, we laid out four problems that are central to the subject: the rate problem, the trigger problem, the energetics problem and the interplay of scales problem. What can we say about these problems and prospects for future progress in reconnection research?

With respect to the rate problem, two paths to fast reconnection have now been identified. One, which applies in systems for which the predicted width of the resistive Sweet–Parker layer is less than the ion skin depth, is two-fluid or kinetic reconnection. Hall effects have been observed through an out-of-reconnection-plane quadrupolar structure in the reconnecting magnetic field in numerical simulations, laboratory experiments and space satellite data. In dedicated reconnection experiments, the reconnection rate is found to increase rapidly as the ratio of the electron mean free path to the scale length increases. This result is attributed to the large Hall electric field in the reconnection layer just outside the electron diffusion layer near the X-point. This provides decisive verification for the presence of two-fluid processes that increase the reconnection rate in collisionless plasmas. However, in recent simulation work on electron–positron pair plasmas where the motions of positrons and electrons cancel the Hall effect, fast reconnection was still observed. A question remains as to how fast reconnection is induced in this kind of plasma, where no Hall effects exist. The second path, which is allowed by MHD reconnection and for which there is extensive computational evidence but little laboratory evidence, is through the plasmoid instability, which breaks up thin current layers into broadened regions with multiple X-points (plasmoids are also formed in collisionless plasmas undergoing fast reconnection). Theoretically, the two paths converge in situations where the plasmoid scale reaches the ion skin depth. This would introduce kinetic effects into MHD reconnection. Characterizing the plasmoid instability in a large laboratory plasma is a goal for future research.

As to the trigger problem, it has long been argued that formation of a thin current sheet, either spontaneously, through an instability, or driven externally by boundary conditions or turbulence, is a prerequisite for fast reconnection (as the Taylor problem shows, current sheet formation may be necessary but not sufficient [35]). Whether such reconnection is fast because the current sheet reaches the two-fluid scale or because it is plasmoid unstable probably depends on properties of the particular system. The observed power law distribution of solar flare energies [10] is a key observation which trigger theories must explain. Developing simulations or experiments large enough to identify a power law from first principles remains a distant but important goal.

There is important recent progress on the energetics problem. A quantitative inventory of magnetic energy conversion during reconnection was carried out in an MRX collisionless reconnection layer with a well-defined boundary. This study concluded that about half the inflowing magnetic energy is converted to particle energy. This differs from the resistive MHD Sweet–Parker theory, according to which the outbound Poynting flux is nil and the inbound Poynting flux is divided equally between Ohmic heating and ion outflows. These results raise the question of whether there is a universal principle for partitioning of converted energy, an important problem for future research.

While there has also been important progress on the scale problem, it remains extremely challenging both experimentally and theoretically. In contrast with laboratory and heliospheric plasma physics, in which two-fluid physics dominates, MHD has been the traditional plasma model of choice in astrophysical plasma physics. While this choice is justifiable when the ratio of global to kinetic scales is large and the ratio of mean free path to plasma scales is small, a major question remains: is it possible to have fast reconnection in MHD, without invoking kinetic processes? Simple arguments show that fast reconnection and a broad outflow with an ‘open scissor’-shaped X-point are closely linked. A fast reconnecting, Petschek-like structure can be obtained in an MHD system when the local resistivity is enhanced as a function of local current density, but this type of anomalous resistivity has yet to be demonstrated in the laboratory. Two-fluid reconnection which is facilitated by Hall effects has an expanding outflow which is an essential feature for fast reconnection. As the drift velocity of electrons with respect to ions becomes maximum near the X-point of two-fluid reconnection, there may be a common physical mechanism between the Petschek model and the two-fluid models. However, it is important to note that, while in Petschek MHD reconnection the field and flow are diverted by shocks, in two-fluid reconnection they are controlled by separatrices. Clarifying the role, if any, of wave–particle interactions as a mechanism for anomalous resistivity is an important future goal.

Studies of two-fluid and kinetic treatments of reconnection, and research of reconnection in turbulent fluids, have forced us to broaden and re-examine the ‘topological’ nature of reconnection. The linkage between the field line velocity **u**_*B*_ introduced in equation (2.2) and the bulk plasma velocity **u** is straightforward for the MHD model and in situations where **u** is analytic. If **u** is understood to be the velocity of the electron fluid, the connection between some type of plasma velocity and the magnetic field velocity can be preserved. However, the full generalized Ohm’s law (equation (5.3)) shows that both the evolution of field lines and their relationship to plasma flow are far more complex. Theories for the breakdown of flux freezing in a stochastic MHD flow also separate issues of fieldline topology (e.g. existence of magnetic X-points) from magnetic field transport relative to the fluid and raise the question of how energy can be exchanged between field and fluid in a turbulent flow. Stellar dynamos operating in regions undergoing turbulent thermal convection could be natural environments for this type of topological reconnection, although of course some degree of flux freezing is necessary for magnetic field amplification in the first place. Whether topological reconnection in a stochastic flow can occur without the transient amplification and growth of Lorentz forces, which might quench or drastically modify the process, is an important question for future research.

Prospects for future progress depend on continued successful innovations in methodology. The combination of laboratory experiments, space plasma measurements and numerical simulations is proving to be especially successful. We have already mentioned identification of the quadrupolar magnetic field in this context, but there are other examples. Recently the electron diffusion region was identified in the magnetospheric plasma by the MMS [116]. This important discovery benefited from the earlier finding by MRX of high-energy deposition to electrons through the perpendicular (w.r.t. *B*) components of electron current near the X-point (§6; figure 9). Another example is the study of the two-fluid reconnection layer with MRX showing conversion of magnetic energy across a region significantly larger than the narrow electron diffusion region previously assumed to be the site of electron energization. A saddle-shaped electrostatic potential profile was measured in the reconnection plane, and the resulting electric field was found to accelerate ions, which are thermalized by remagnetization in the downstream region. Evidence for the same potential profile and fast ions has been observed in space plasmas.

As of this writing, the space–laboratory connection is stronger than the astrophysical–laboratory connection. This is true in part because MHD reconnection is more difficult to study in the laboratory than two-fluid or collisionless reconnection. Clarifying the role of kinetic processes in reconnection in astrophysical systems in which the global scales are well described by MHD is a key problem in which experiment, simulation and theory could mutually motivate and reinforce one another. Certain regimes of astrophysical interest are being accessed by current high-energy density plasma reconnection experiments. This is a promising area for the future.

Many of the examples and problems discussed here demonstrate the necessity of investigating reconnection dynamics beyond the idealized classical single quasi-stationary X-line geometry or Sweet–Parker model, and exploring the recently discovered more realistic, high-dynamic reconnection regimes characteristic of large systems, such as those found in most space and astrophysical environments. These complex regimes feature multiple X-lines, plasmoid and flux-rope formation due to secondary instabilities and the self-consistent emergence of turbulence and accompanying coherent structures under a variety of plasma conditions. This theme has emerged in the last several years as the new paradigm of how magnetic reconnection really happens in natural plasmas. Understanding the generation and influence of secondary reconnection instabilities is one of the primary goals of two new reconnection experiments. Accordingly, a key part of new kinetic simulation efforts will be directed at modelling these devices in order to validate our codes and test theoretical ideas. These developments will enable us to make better predictions regarding space and astrophysical plasmas. With improved understanding of reconnection as a basic process, it will become possible to sharpen observational diagnostics, develop accurate subgrid models of reconnection to use in large-scale computations and focus reconnection research on the specialized features of natural plasmas throughout the Universe.

## Footnotes

1 Ohm’s law in its most general form is the momentum equation for the electron fluid. In MHD, the electric field force measured in the fluid rest frame and the collisional drag force, which is the source of resistivity, are balanced.

2 It is possible to circumvent this conservation relation in special cases, e.g. by recombination of plasma in weakly ionized gases [18]

3 In a cylinder of length 2*πR*, *q*≡*rB*_*z*_/*RB*_*ϕ*_, where *r* is the distance from the axis; a field line winds by an angle 2*π*/*q* over the length of the cylinder.

4 The current sheet solution was recently confirmed by an innovative numerical method [36].

5 The ion skin depth is the gyroradius of an ion with velocity *v*_A_ and can also be expressed in terms of the ion plasma frequency *ω*_pi_≡(4*πn*_i_*e*^2^/*m*_i_)^1/2^ as *c*/*ω*_pi_.

6 If there is a field component perpendicular to the reconnection plane—a guide field—the ions are tied to the magnetic field on the Larmor scale *r*_i_ and the two-fluid threshold is *δ*∼*r*_i_.

## Appendix A. Experimental devices dedicated to study of magnetic reconnection

A series of dedicated laboratory experiments have been carried out to study the fundamental processes of reconnection by making a reconnecting current sheet in a controlled manner. In these experiments, a reconnection layer can be created by driving oppositely directed field lines into the neutral sheet generating a reconnection region in a controlled setting with varying plasma parameters.

Table 1 summarizes major devices dedicated to the study of the physics of magnetic reconnection. Here we highlight four dedicated reconnection devices which generated results discussed in this review.

(1) TS-3/4 device. In the Todai spheromak-3/4 facility [117], two spheromak-type plasma toroids merged together, contacting and connecting along a toroidally symmetric line. The two toroidally shaped spheromaks, carrying equal toroidal current with the same or the opposite toroidal field, are forced to merge by controlled external coil currents. These are called co-helicity merging or counter-helicity merging, respectively. A strong dependence of the reconnection speed on the merging angle of field lines and the global forcing was observed
(2) SSX facility. The Swarthmore Spheromak Experiment (SSX) [12] facility studies magnetic reconnection also through the merging of spheromaks. Reconnection physics, particularly its global characteristics, has been studied in a number of geometries. Different types of flux-conserving conductors consisting of two identical copper containers have been used. Merging of a pair of counter-helicity spheromaks generates turbulent 3D magnetic reconnection dynamics at the mid-plane.
(3) MRX device. MRX was built at PPPL in 1995 [4,9] to investigate the fundamental physics of magnetic reconnection. The analysis focuses on the coupling between local features of the reconnection layer and global properties such as the external driving force and the evolution of plasma equilibrium. In addition to the results reported here, the recent experimental work of MRX covers reconnection phenomena in space astrophysical plasmas such as (i) asymmetric reconnection in which one side of the inflowing plasma density is significantly higher than that of the other side, (ii) guide field reconnection, (iii) reconnection in partially ionized plasmas, and (iv) flux rope reconnection relevant to solar flares.
(4) VTF facility. The versatile toroidal facility (VTF) reconnection experiment [13] was developed at the Massachusetts Institute of Technology. The VTF explored fast magnetic reconnection in collisionless plasma for a configuration with a strong variable guide magnetic field. The understanding gained from research on reconnection in the VTF was applied to an interpretation of *in situ* measurements of the electron phase space distribution during reconnection in the deep magneto tail. This is of particular relevance to the reconnection event observed by the WIND satellite.

**Table 1.** Experimental devices dedicated to the study of magnetic reconnection.

device	location	year built	experimentalists	geometry and references cited in the text
3D-CS	Russia	1970	Syrovatskii, Frank	linear [4,40]
LPD, LAPD	UCLA, Los Angeles, CA	1980	Stenzel, Gekelman, Carter	linear [3,4]
TS-3/4	Tokyo, Japan	1988	Katsurai, Ono	toroidal, merging [3,4,11,117]
MRX	Princeton University, Princeton, NJ	1995	Yamada, Ji	toroidal, merging [3,4,9,30–32,65,84,85,94–96,103,110,112,118]
SSX	Swarthmore College, Swarthmore, PA	1996	Brown	toroidal [4,12,91]
VTF	MIT, Cambridge, MA	1998	Fasoli, Egedal	toroidal [4,13]
laser-driven	USA, UK, China	2006	Nilson, Li, Zhong, Dong, Fox, Fiksel	planar [119]
merging				
VINETA II	Max-Planck Institute, Munich, Germany	2012	Grulke, Klinger	linear [120]
TREX	University of Wisconsin-Madison, Madison, WI	2013	Egedal, Forest	toroidal [107]
FLARE	Princeton Plasma Physics Laboratory, Princeton, NJ	2017	Ji *et al.* (to be commissioned in 2017)	toroidal [106,108]
